# Bowel Dysfunction After Colon Cancer Surgery: A Prospective, Longitudinal, Multicenter Study

**DOI:** 10.1097/DCR.0000000000003358

**Published:** 2024-06-20

**Authors:** Sofia J. Sandberg, Jennifer M. Park, Viktor A. Tasselius, Eva Angenete

**Affiliations:** 1 Department of Surgery, Scandinavian Surgical Outcomes Research Group, Institute of Clinical Sciences, Sahlgrenska Academy, University of Gothenburg, Gothenburg, Sweden; 2 Department of Surgery, Region Västra Götaland, Sahlgrenska University Hospital/Östra, Gothenburg, Sweden; 3 Department of Public Health and Community Medicine, Institute of Medicine, Sahlgrenska Academy, University of Gothenburg, Gothenburg, Sweden

**Keywords:** Colon cancer, Functional outcome, Low anterior resection syndrome

## Abstract

**BACKGROUND:**

Longitudinal studies on functional outcomes after colon resection are limited.

**OBJECTIVE:**

To evaluate bowel dysfunction and related distress 1 and 3 years after colon resection using the low anterior resection syndrome score as well as specific validated items.

**DESIGN:**

This study presents the long-term results of bowel dysfunction and related distress based on the Quality of Life in Colon Cancer study, an observational, prospective multicenter study of patients with newly diagnosed colon cancer.

**SETTINGS:**

The study was conducted at 21 Swedish and Danish surgical centers between 2015 and 2019.

**PATIENTS:**

All patients who underwent right-sided or left-sided colon resection were considered eligible. Exclusion criteria were age younger than 18 years, cognitive impairment, or inability to understand Swedish/Danish. Patients completed extensive questionnaires at diagnosis and after 1 and 3 years. Clinical data were supplemented by national quality registries.

**MAIN OUTCOME MEASURES:**

The low anterior resection syndrome score, specific bowel symptoms, and patient-reported distress were assessed.

**RESULTS:**

Of 1221 patients (83% response rate), 17% reported major low anterior resection syndrome 1 year after either type of resection; this finding was consistent at 3 years (17% right, 16% left). In the long-term, the only significant difference between types of resections was a high occurrence of loose stools after right-sided resections. Overall, less than one-fifth of patients experienced distress, with women reporting more frequent symptoms and greater distress. In particular, incontinence and loose stools correlated strongly with distress.

**LIMITATIONS:**

Absence of prediagnosis bowel function data.

**CONCLUSIONS:**

Our study indicates that bowel function remains largely intact after colon resection, with only a minority reporting significant distress. Adverse outcomes were more common among women. The occurrence of loose stools after right-sided resection and the association between incontinence, loose stools, and distress highlights a need for postoperative evaluations and more thorough assessments beyond the low anterior resection syndrome score when evaluating patients with colon cancer. See the **Video Abstract**.

**DISFUNCIONAMIENTO INTESTINAL DESPUÉS DE LA CIRUGÍA POR CÁNCER DE COLON: ESTUDIO PROSPECTIVO, LONGITUDINAL Y MULTICÉNTRICO:**

**ANTECEDENTES:**

Los estudios longitudinales sobre el resultado funcional después de una resección cólica son limitados.

**OBJETIVO:**

Examinar la disfunción intestinal y el malestar relacionado uno y tres años después de la resección del colon utilizando la puntuación de referencia en el síndrome de resección anterior baja (LARS), así como otros ítems de validez específica.

**DISEÑO:**

Este estudio presenta los resultados a largo plazo de la disfunción intestinal y la angustia relacionada según el estudio QoLiCOL (Quality of Life in COLon cancer), un analisis observacional, prospectivo y multicéntrico de pacientes con cáncer de colon recién diagnosticado.

**AJUSTES:**

El presente estudio fué realizado en 21 centros quirúrgicos suecos y daneses entre 2015 y 2019.

**PACIENTES:**

Todos los pacientes sometidos a resección de colon, tanto del lado derecho como el izquierdo se consideraron elegibles. Los criterios de exclusión fueron tener menos de 18 años, deterioro cognitivo o incapacidad para entender sueco/danés. Los pacientes completaron extensos cuestionarios en el momento del diagnóstico y después de uno y tres años. Los datos clínicos se complementaron con los registros de calidad binacionales.

**PRINCIPALES MEDIDAS DE RESULTADO:**

Se evaluaron los síntomas intestinales específicos, la puntuación LARS y la angustia manifestada por cada paciente.

**RESULTADOS:**

De 1221 pacientes (tasa de respuesta del 83%), el 17% informó LARS mayor un año después de cualquier tipo de resección, consistente a los tres años (17% derecha, 16% izquierda). A largo plazo, la única diferencia significativa entre los tipos de resección fue una alta incidencia de heces liquidas después de las resecciones del lado derecho. En general, menos de una quinta parte de los pacientes experimentaron angustia, y fué la poblacion femenina quién informó de síntomas más frecuentes y de mayor angustia. En particular, la incontinencia y las heces liquidas se correlacionaron fuertemente con la angustia.

**LIMITACIONES:**

Ausencia de datos de función intestinal previos al diagnóstico.

**CONCLUSIONES:**

Nuestro estudio indica que la función intestinal permanece en gran medida intacta después de la resección del colon, y sólo una minoría reporta malestar significativo. Los resultados adversos fueron más comunes en la población femenina. La aparición de heces liquidas después de la resección del lado derecho y la asociación entre incontinencia, heces liquidas y malestar resalta la necesidad de evaluaciones postoperatorias y valoraciones más exhaustivas más allá de la puntuación LARS al evaluar a los pacientes con cáncer de colon. *(Traducción—Dr. Xavier Delgadillo*)

Colorectal cancer ranks among the most prevalent types of cancer.^1^ Treatment with curative intent often includes surgical resection of the engaged bowel segment followed by anastomosis. For many patients with rectal cancer, the removal of the rectum leads to impaired bowel function, referred to as low anterior resection syndrome (LARS).^2,3^ The functional impairments after rectal cancer surgery are well documented^4,5^; however, bowel function after surgery for colon cancer has not been studied to a similar extent.

Most available studies have consisted of relatively small patient cohorts with a retrospective or a cross-sectional design. A recent meta-analysis was limited to a descriptive analysis of symptoms over time, and differences between right-sided and left-sided resections were unable to be addressed because of data heterogeneity and a shortage of longitudinal studies.^6^ Some studies, however, have suggested that after left or sigmoid resection, patients mainly experience symptoms associated with constipation, whereas patients treated with right-sided resection frequently report increased bowel movements, loose or liquid stools, fecal urgency, and incontinence.^7–11^ It has also been proposed that colon resection minimally affects bowel function, as functional outcomes have been found comparable with those of the general population.^12^

The Quality of Life in Colon Cancer (QoLiCOL) study is an observational, prospective, longitudinal, multicenter study of functional outcome and QoL among patients treated for colon cancer.

Our objectives were to compare bowel function and associated distress between right-sided and left-sided resections 1 and 3 years after treatment. We anticipated symptom variance based on resection type and expected improvement over time. We also theorized that symptoms such as “urgency” and “clustering” would be most distressing given their significant impact on QoL in the LARS score, which is frequently used in rectal cancer evaluations.^13^

## MATERIALS AND METHODS

### Study Design and Participants

This study includes all patients from the QoLiCOL study who underwent a colon resection with anastomosis. The study was registered at clinicaltrials.gov (NCT02530593) and conducted between 2015 and 2019 at 21 surgical units in Sweden and Denmark. All patients with a verified adenocarcinoma, irrespective of staging or planned treatment, were considered eligible for the study. Exclusion criteria were age younger than 18 years, cognitive impairment, or language barriers. Patients were invited to participate after the diagnosis but before treatment commencement. After the agreement to participate, the recruiting hospital reported the patient to the study secretariat. Thereafter, the research nurses at the study secretariat administered all further communication with the included patients (making telephone calls; sending and receiving questionnaires, reminders, and thank-you notes). The implementation of a somewhat intense follow-up strategy is a strategy used by our research group that has been reported to improve the response rate.^14^ Questionnaires were sent to patients at diagnosis and after 1 and 3 years. Clinical data were sourced from national quality registries.^15,16^

In the current study, we excluded patients with a permanent stoma and those who underwent total colectomy, as well as a few patients who had been operated on only with a short palliative resection of the transverse colon. Patients were categorized by oncological resection type: right-sided (ileocecal or right colon) or left-sided (left colon or sigmoid). Ethical approval was obtained from the Regional Ethical Review Boards in Gothenburg, Sweden (registration no. EPN 957-14) and Denmark (registration no. H-16027323).

### Questionnaires

The questionnaires in the QoLiCOL study consisted of roughly 250 inquiries addressing various aspects, including health-related QoL and functional limitations. Additional questions on frequency, intensity, and duration assessed physical symptoms. The questionnaires were constructed on the basis of qualitative interviews combined with questions that had been validated previously. Content validation was assessed by an expert panel (colorectal surgeons, oncologists, gynecologists, and nurses), followed by face-to-face validation by patients with colon cancer using previously described methods.^17^ Not all questions were assessed within the scope of this study. Bowel function was assessed by specific questions in addition to the validated LARS score.^13^

The LARS score encompasses 5 questions on bowel function: incontinence for flatus, incontinence for liquid stool, frequency of bowel movements, clustering, and urgency. Depending on the symptom frequency, the total score ranges from 0 to 42, divided into 3 categories: no LARS (0–19 points), minor LARS (20–29 points), or major LARS (30–42 points).

The score has been validated for rectal cancer but not for colon cancer.^13,18^ Therefore, our questionnaire also included additional questions on bowel function, such as leakage of solid stools, occurrence of loose stools, and use of antidiarrheal medication or laxatives. The questionnaires also featured an anchoring question on distress, phrased as “If you were to live the rest of your life with your bowel problems, as they have been for the last month, how would you experience it?” with response options of “no,” “little,” “some,” or “much” distress.

### Definitions

The total LARS score was calculated using major LARS as the outcome measure. Each separate item in the LARS score, as well as the other questions on bowel function in the questionnaire, were analyzed. A frequency of “more than 1 per week” was considered clinically significant, allowing for dichotomization of answers. Regarding the question “frequency of bowel movements,” a frequency of more than 4 times per day or less than 1 per day was considered a significantly abnormal frequency. Patients were asked whether they had used antidiarrheal medication or laxatives in the past month (yes/no). The distress related to the bowel dysfunction was dichotomized to “no” (no/little distress) and “yes” (some/much distress).

### Statistical Analysis

Prior to analyses, a statistical analysis plan was created. A power calculation had previously been made for the QoLiCOL study, targeting a study cohort of 1500 patients with the subgroups: palliative treatment (20%–25%), curative surgery (40%–45%), and curative surgery followed by adjuvant treatment (25%–30%). This would allow for the estimation of the prevalence of health-related factors, with differences between groups down to 15% and a statistical power of 80%. This calculation was based on binomial distribution approximation with a prevalence of 50% and a significance level of 5%.

Patient characteristics of responders (response to the questionnaires at baseline and at least 1 time point during follow-up) and nonresponders were reported separately. Differences in symptoms between right-sided and left-sided resections were evaluated, with an additional categorization based on sex, using the χ^2^ test. To assess the longitudinal course of major LARS and distress, a generalized linear mixed-effects model with a logit link was used to account for the repeated-measurements over time. Time, age (younger than 70 years or 70 years and older), sex, and type of resection were considered as fixed effects as well as 2- and 3-way interaction effects. Random effects were used to account for the intrapatient dependence. All patients experiencing distress, regardless of their LARS score, were included in the model. The results were reported as OR and 95% CIs.

A similar approach was used in the analyses of the association between specific symptoms of bowel dysfunction and distress. Interaction terms between symptoms and time were added to explore how the effect of each symptom might change over time. Before the combined model, we conducted separate analyses for each symptom, followed by an assessment of multicollinearity within the combined model to ensure the independence of explanatory variables. Sex and age were considered as potential confounders and were adjusted for. The symptom “incontinence for solid stools” had few events and was joined with “incontinence for liquid stools” to facilitate analysis.

The observed prevalence for 1 and 3 years was presented with a 95% CI. Because not all participants responded in both years, we also calculated the prevalence for each symptom and distress for patients with responses at both time points (complete cases only) and through imputation methods: last observation carried forward and next observation carried back. There were no significant differences for any symptom across these methods, so the observed prevalence was reported.

A *p* value of <0.05 was considered statistically significant. Statistical analyses were performed using R version 3.4.3 (R Foundation for Statistical Computing).^19^ The results were reported according to the Strengthening the Reporting of Observational Studies in Epidemiology guidelines.^20^

## RESULTS

**FIGURE 1.**
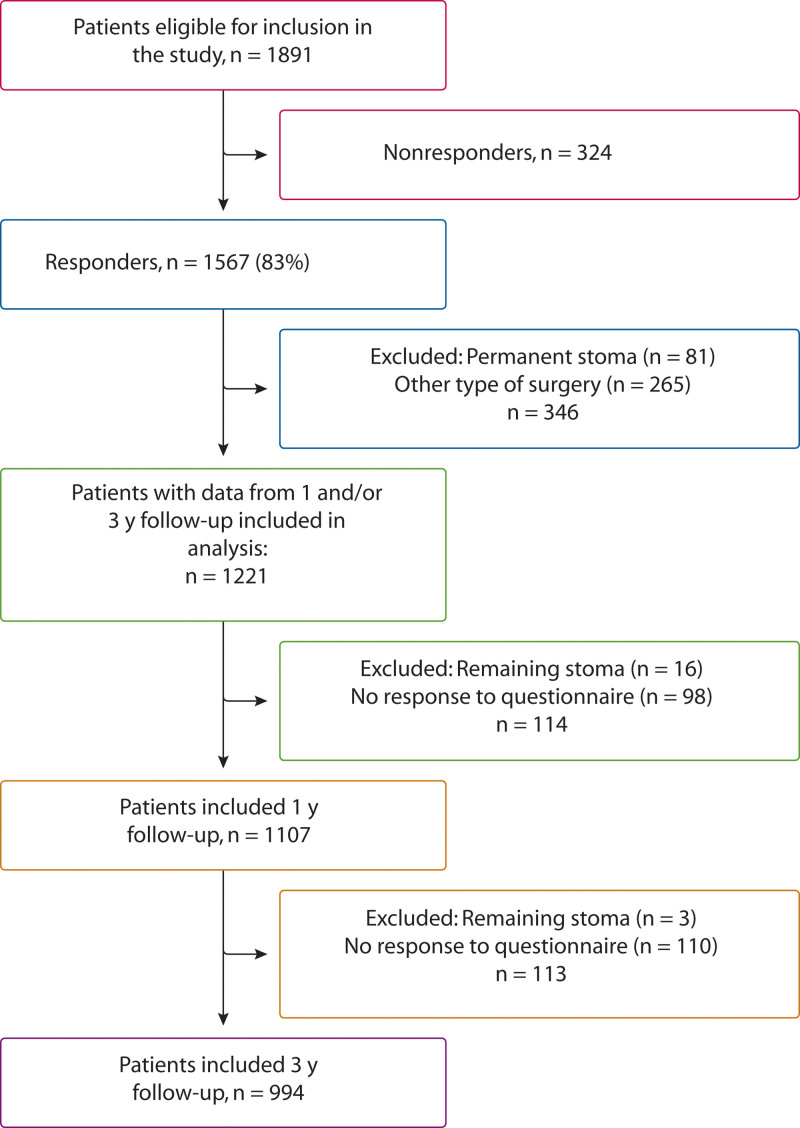
Flow chart of patient inclusion and exclusion.

Between 2015 and 2019, informed consent was obtained from 1891 patients who were considered eligible for the QoLiCOL study. A total of 1567 patients (83%) completed the questionnaires at baseline and at least 1 time point during follow-up. In the current study, we excluded 81 patients with a permanent or persistent stoma during follow-up as well as 265 patients who had undergone surgery not possible to categorize into right-sided or left-sided resection (Fig. 1).

Accessible to analyses were 1221 patients with data from at least 1 follow-up. Of these, 1107 patients had data from the 1-year follow-up, and 994 had data from the 3-year follow-up. For 880 patients, data were accessible from both follow-ups.

### Patient Characteristics

Among the included patients, 754 (62%) underwent right-sided resection (Table 1). A total of 629 patients (52%) were men, and the median age among included patients was 72 years. There were more men who operated on left-sided resection, and patients in this group were slightly younger (median age 70 versus 74 years), had a statistically significant lower ASA classification, and had less advanced tumor stage.

**TABLE 1.** Patient characteristics and surgical variables

*Variable*	*Overall (N = 1221*)	*Right-sided resection (N = 754 [62%]*)	*Left-sided resection (N = 467 [38%]*)	*p*
Sex				**0.006**
Men	629 (52)	365 (48)	264 (57)	
Women	592 (48)	389 (52)	203 (43)	
Age, y	72.0 (65.0–78.0)	74.0 (67.0–79.0)	70.0 (64.0–76.0)	**<0.001**
BMI	25.7 (23.5–28.7)	25.7 (23.5–28.7)	25.8 (23.5–28.7)	0.6
Missing	11	10	1	
ASA				**<0.001**
I	161 (14)	88 (12)	73 (16)	
II	731 (62)	430 (59)	301 (66)	
III	282 (24)	209 (28)	73 (16)	
IV	13 (1)	7 (1)	6 (1)	
Missing	34	20	14	
Tumor stage (UICC)				**0.002**
I	264 (26)	158 (25)	106 (27)	
II	250 (24)	143 (22)	107 (28)	
III	441 (43)	301 (47)	140 (36)	
IV	70 (7)	35 (6)	35 (9)	
Missing	196	117	79	
Neoadjuvant chemotherapy	18 (1.5)	9 (1.2)	9 (1.9)	0.3
Missing	1	0	1	
Minimally invasive surgery	548 (45)	335 (44)	213 (46)	0.7
Missing	1	0	1	
Setting				0.6
Elective	1217 (100)	752 (100)	465 (100)	
Emergency	4 (0.3)	2 (0.3)	2 (0.4)	
Temporary stoma	31 (2.5)	7 (0.9)	24 (5.1)	**<0.001**
Missing	1	1	0	
Planned adjuvant chemotherapy	123 (37)	72 (37)	51 (38)	0.9
Missing	888	557	331	

Data are presented as n (%) or median (interquartile range).

UICC = Union for International Cancer Control.

Nonresponders showed a similar distribution to responders in terms of sex, BMI, and tumor stage but were slightly older (median age 76 years) and had a higher ASA classification (ASA III/IV 41% compared to 25%; see Supplemental Table 1 at http://links.lww.com/DCR/C356). Furthermore, nonresponders were less likely to undergo minimally invasive surgery (35% vs 45%) and more often required emergency operations.

### Symptoms of Bowel Dysfunction

**FIGURE 2.**
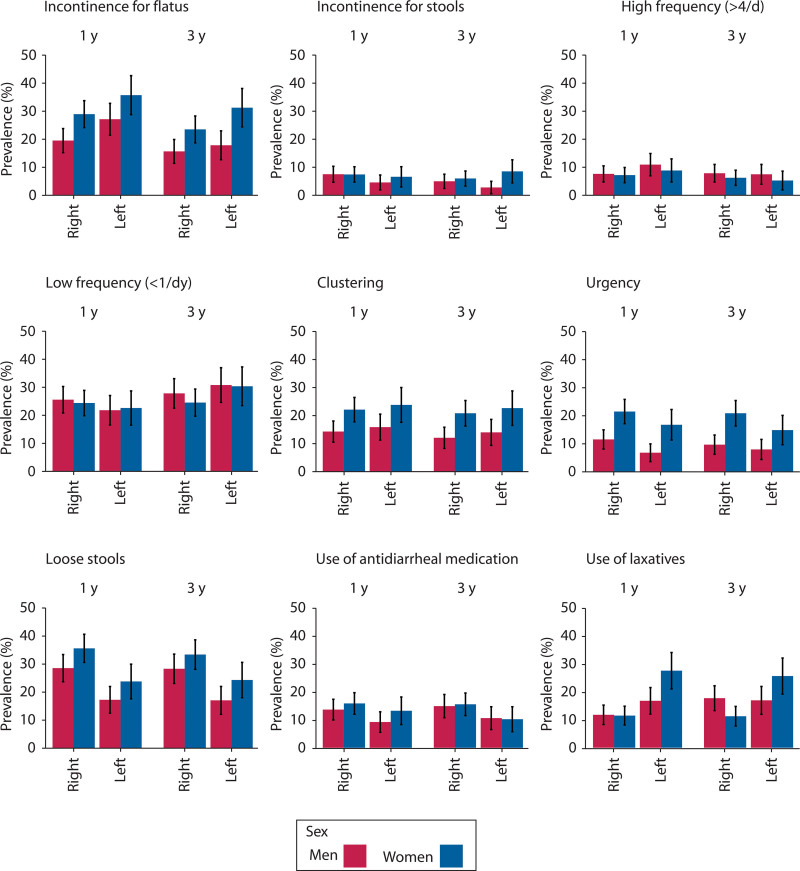
Bar charts of the specific symptoms of bowel dysfunction occurring more frequently than 1 time per week 1 and 3 y after right-sided or left-sided resection.

The specific symptoms of bowel dysfunction occurring more often than weekly are shown in Figure 2 (statistics provided in Supplemental Table 2 at http://links.lww.com/DCR/C357). One year after surgery, urgency and loose stools were significantly more common after right-sided resection (17% versus 11%, *p* = 0.01, and 32% versus 20%, *p* < 0.001), whereas leakage of flatulence was significantly more common after left-sided resection (30% versus 24%, *p* = 0.02).

Three years after surgery, no differences between the types of resections were statistically significant except for loose stools, which were more common after right-sided resection (31% versus 20%, *p* < 0.001).

### Differences Between Men and Women

Women generally reported worse bowel function. After 3 years, symptoms such as incontinence for flatus, as well as clustering and urgency, were significantly more common among women than men, regardless of resection type.

After right-sided resection, 24% of women and 16% of men reported incontinence for flatus (*p* = 0.02). After left-sided resection, the figures were 31% for women and 18% for men (*p* = 0.003). Clustering was reported by 21% of women after right-sided resection and by 23% after left-sided resection, whereas the corresponding figures for men were 12% and 14%, respectively (*p* = 0.006 and *p* = 0.04). Twice as many women reported urgency, irrespective of the resection type, 21% versus 10% (*p* < 0.001) after right-sided resection and 15% versus 8% (*p* = 0.04) after left-sided resection.

Few patients (<10%) reported incontinence for stools with little difference between sexes and resection types. The only significant difference was noted 3 years after left-sided resection (9% women versus 3% men, *p* = 0.02). There were no significant differences between the sexes in terms of frequency of bowel movements or the occurrence of loose stools.

### Use of Antidiarrheal Medication and Laxatives

The use of antidiarrheal medication was more common after right-sided resection, with a significant difference after 3 years (15% versus 10%, *p* = 0.04). Laxatives were overall significantly more common after left-sided resection during the follow-up after 3 years (21% versus 14%; *p* = 0.001; see Supplemental Table 2 at http://links.lww.com/DCR/C357).

### Prevalence of Major LARS

**FIGURE 3.**
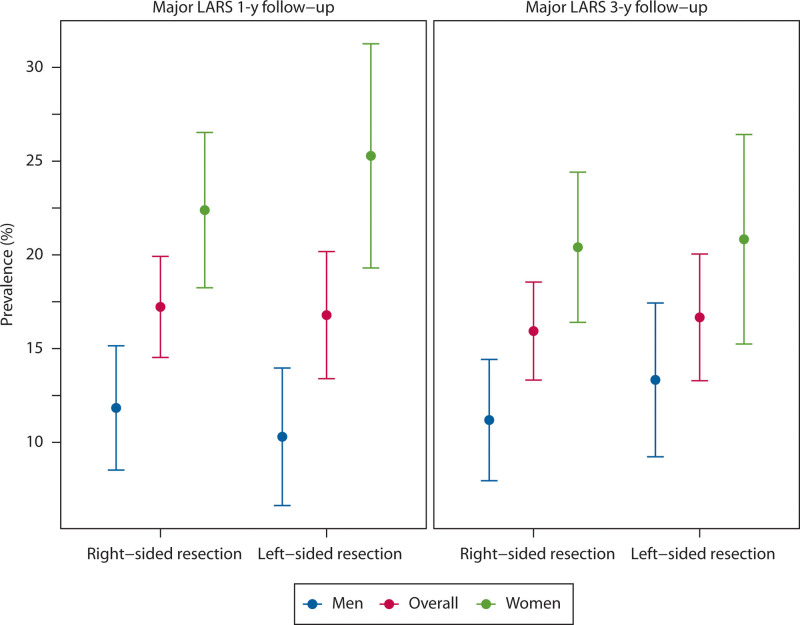
The observed prevalence of major LARS 1 and 3 y after right-sided and left-sided colonic resection. Data are shown for men, women, and the overall patient group. LARS = low anterior resection syndrome.

Figure 3 illustrates the observed prevalence of major LARS. After a 1-year follow-up, 17% reported major LARS after either type of resection, and this finding was consistent at 3 years (17% right-sided resection, 16% left-sided resection). Major LARS was about twice as common among women after both right-sided and left-sided resection. One year after right-sided resection, 22% of women reported major LARS compared to 12% of men (*p* < 0.001). After left-sided resection, 25% of women reported major LARS compared to 10% of men (*p* < 0.001).

The statistical analysis of the longitudinal course of major LARS indicated that there was no improvement over time and that sex, age, or type of operation had no significant association with the prevalence of major LARS over time (see Supplemental Table 3 at http://links.lww.com/DCR/C358).

### Distress Related to Bowel Function

**FIGURE 4.**
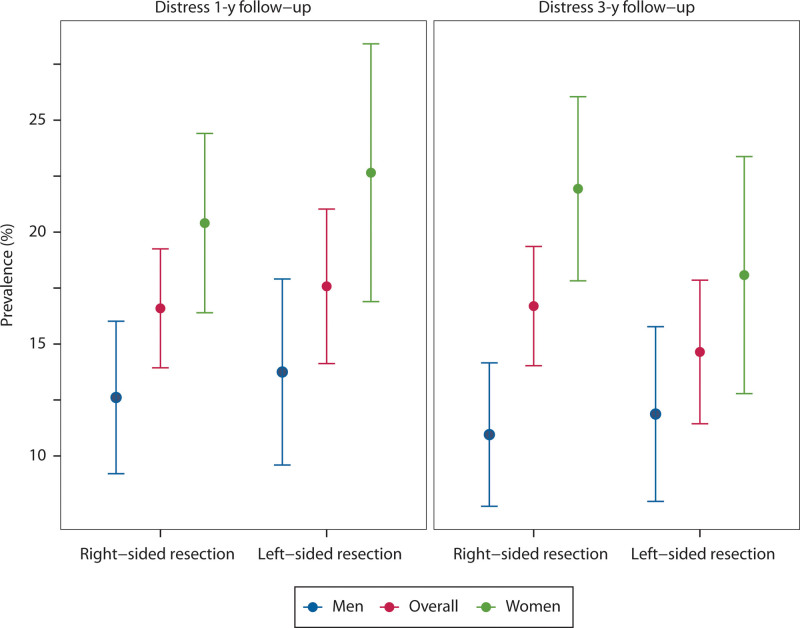
The observed prevalence of distress 1 and 3 y after right-sided and left-sided colonic resection. Data are shown for men, women, and the overall patient group.

Overall, less than one-fifth of patients experienced distress related to bowel function (Fig. 4). Regardless of resection type, women more often reported distress. One year after right-sided resection, 20% of women versus 13% of men felt distress (*p* = 0.009), and after left-sided resection, 23% of women and 14% of men felt distress (*p* = 0.02). When limiting analysis to the patients with major LARS, approximately half of the patients reported distress related to bowel dysfunction.

Statistical analysis of distress showed no improvement over time. Factors such as sex, age, or the type of resection did not demonstrate any significant association with the prevalence of distress over time (see Supplemental Table 3 at http://links.lww.com/DCR/C358).

### Specific Symptoms Associated With Distress

Incontinence for flatus (OR 4.75, 95% CI, 2.04–11.03; *p* < 0.001), incontinence for stools (OR 5.38; 95% CI, 1.28–22.64; *p* = 0.022), clustering (OR 4.78; 95% CI, 1.61–14.25; *p* = 0.004), and occurrence of loose stools (OR 7.00; 95% CI, 2.72–18.04; *p* < 0.001) were all symptoms found to be significantly associated with distress (see Supplemental Table 4 at http://links.lww.com/DCR/C359).

The correlation between these symptoms and distress persisted over time, except for clustering, which demonstrated a diminished association with distress from 1 to 3 years after surgery.

## DISCUSSION

In the current study, we present longitudinal data on bowel function and distress based on the QoLiCOL study. We observed with interest that most patients reported unaffected bowel function after colonic resection, regardless of the side of resection, and that only a minority of patients experienced distress related to their bowel function.

Overall, women experienced worse bowel function and higher levels of distress compared to men. The findings of impaired bowel function among women align with the general population, and our observed prevalence of major LARS did not differ remarkably from some previous analyses on normative data, showing a prevalence of major LARS among men of approximately 7% to 18% and among women up to 25%, depending on age group.^21–24^ A recent meta-analysis of functional outcome after surgery for colon cancer indicated a pooled prevalence of major LARS of 21% with no difference in terms of time to follow-up or type of colectomy.^6^

We found a high prevalence of weekly leakage of flatulence, reported by 36% of women 1 year after left-sided resection and 31% after 3 years. These results are comparable with findings from the general populations in Denmark and the Netherlands, and they also parallel earlier studies by our research team on a Swedish demographic population.^21,22,25^ Our results regarding incontinence for stools, high frequency of bowel movements, clustering, and urgency were also comparable with previously reported bowel function of the normal population.^21,22^

Our findings suggest that the majority of symptoms observed after colon surgery are likely attributable to preexisting bowel dysfunction rather than being direct consequences of the surgical procedure itself. Loose stools have been reported in approximately 6% to 13% of the general population.^8,26,27^ However, our cohort found an overall high prevalence (28%–35%) of loose stools after right-sided resection among both sexes, and this finding persisted over time. The difference supports the idea that loose stools are related to the surgical procedure. These results are consistent with prior findings of loose stools after right-sided resection, possibly because of bile acid malabsorption.^8,9,28,29^ Despite the high prevalence of loose stools, it did not seem to correspond to an increased frequency of bowel movements.

We found that loose stools, as well as incontinence symptoms, had a strong association with distress. Loose stools after right-sided resection have previously been reported to be associated with a decline in QoL.^8^ There was a clear mismatch between symptom and treatment, especially between antidiarrheal and loose stools. Half of the patients with loose stools did not use antidiarrheal medication, suggesting potential underdiagnosis or undertreatment.

To our knowledge, this is the first longitudinal study with a follow-up period of 3 years of bowel function and distress in an unselected cohort of patients after colonic resection. Our study’s strengths lie in its longitudinal design, multicenter approach, relatively large population size, and inclusion regardless of tumor stage or planned treatment. A high response rate minimizes the risk of selection bias, and the comparison between responders and nonresponders showed fairly comparable groups, indicating that our results can possibly be extrapolated to a larger community. A possible limitation is the lack of evaluation of normal bowel function before diagnosis of the included cohort. However, such information is difficult to obtain because altered bowel function is often expected in patients with newly diagnosed, and yet not treated, colorectal cancer.

The dichotomization of response options regarding frequency represents a potential limitation. We selected a threshold of “more than 1 per week,” which is somewhat restrictive. We considered this cutoff as clinically relevant, especially when the aim was to capture the association with distress. This cutoff aligns with the scoring methodology used in the LARS score, where it results in the highest item scores, reflecting significant clinical impact and effect on QoL.^13^ However, the use of many nonvalidated bowel function scores with different definitions has made comparisons between studies difficult. The prevalence of urgency, for example, varies in the literature from 16% to 77% after right-sided resection and between 10% and 81% after left-sided, depending on the definition.^7,30^ The LARS score has been extensively validated for evaluating bowel dysfunction after resection for rectal cancer, and although it can be used to estimate bowel dysfunction in the general population as well as other patient groups, our results highlight that the score does not cover all aspects of bowel dysfunction relevant for patients who have undergone operations for colon cancer.

## CONCLUSIONS

Our study indicates that bowel function remains largely intact after colon resection, as the prevalence of most symptoms of dysfunction is comparable to those previously reported from the general population. Although we found modest impairment in bowel function after colon resection, with a minority experiencing distress, sex-specific differences were observed, with women being more adversely affected. The prevalence of loose stools after right-sided resections and the strong correlation between incontinence symptoms and distress underscore the need for comprehensive postoperative evaluations beyond the LARS score. Early diagnosis and intervention are important, especially targeting symptoms such as loose stools and incontinence.

## Supplementary Material


