# Supplementary material for: Bowel Dysfunction After Colon Cancer Surgery: A Prospective, Longitudinal, Multicenter Study
Source: Dis Colon Rectum. 2024 Jun 20;67(10):1322–31. doi: 10.1097/DCR.0000000000003358 (PMC11373893; doi:10.1097/DCR.0000000000003358)
Supplement: Supplementary file 2 [file dcr-67-1322-s002.pdf]

Supplement Table 1. Comparison responders versus non-responders.

Variable	Responders<br>N = 1,221	Non-responders<br>N = 324	p value
Sex			0.4
Men	629 (52)	159 (49)	
Women	592 (48)	165 (51)	
Age (years)	72.0 (65.0 - 78.0)	76.0 (69.0 - 81.0)	**<0.001**
BMI (kg/m^2)**	25.7 (23.5 - 28.7)	25.8 (23.4 - 29.7)	0.5
Missing	*11 (1)*	*2 (1)*	
ASA***			**<0.001**
I	161 (14)	24 (7.7)	
II	731 (62)	163 (52)	
III	282 (24)	112 (36)	
IV	13 (1.1)	14 (4.5)	
Missing	*34 (3)*	*11 (3)*	
Tumor stage (UICC)****			0.2
I	264 (26)	66 (25)	
II	250 (24)	60 (22)	
III	441 (43)	114 (42)	
IV	70 (6.8)	29 (11)	
Missing	*196 (16)*	*55 (17)*	
Neoadjuvant chemotherapy	18 (1.5)	6 (1.9)	0.6
Missing	*1 (0)*	*2 (1)*	
Minimally invasive surgery	548 (45)	115 (35)	**0.002**
Missing	*1 (0)*		
Setting			**0.008**
Elective	1,217 (100)	318 (98)	
Emergency	4 (0.3)	6 (1.9)	
Temporary stoma	31 (2.5)	13 (4.0)	0.2
Missing	1	1	
Planned adjuvant chemotherapy	123 (37)	28 (28)	0.11
Missing	*888 (73)*	*225 (69)*	

*n (%); Median (IQR)

**BMI = Body Mass Index

***ASA = The American Society of Anesthesiologists (ASA) physical status classification

****UICC = The Union for International Cancer Control (UICC)
