# Supplementary material for: Bowel Dysfunction After Colon Cancer Surgery: A Prospective, Longitudinal, Multicenter Study
Source: Dis Colon Rectum. 2024 Jun 20;67(10):1322–31. doi: 10.1097/DCR.0000000000003358 (PMC11373893; doi:10.1097/DCR.0000000000003358)
Supplement: Supplementary file 3 [file dcr-67-1322-s003.pdf]

Supplemental Table 2. Prevalence of symptoms of bowel dysfunction total

	1 year			*3 years*		
Variable	Right-sided resection (%)	Left-sided resection (%)	p	Right-sided resection (%)	Left-sided resection (%)	p
Incontinence for flatus	24.3 (21.1-27.6)	30.1 (26.4-35.3)	**0.022**	19.7 (16.5-23.0)	23.9 (19.7-28.1)	0.139
Incontinence for stools	7.5 (5.5-9.4)	5.5 (3.3-7.6)	0.238	5.5 (3.7-7.4)	5.4 (3.1-7.6)	1
High frequency (>4/day)	7.4 (5.4-9.4)	10.0 (7.2-12.9)	0.158	7.0(4.9-9.1)	6.5 (4.0-9.0)	0.856
Low frequency (<1/day)	25.0 (21.7-28.3)	22.2 (18.2-26.2)	0.325	26.2 (22.6-29.7)	30.6 (26.0-35.3)	0.146
Clustering	18.3 (15.4-21.2)	19.2 (15.5-23.0)	0.745	16.6 (13.6-19.6)	17.9 (14.1-21.6)	0.664
Urgency	16.5 (13.7-19.3)	11.0 (8.0-14.0)	**0.014**	15.4 (12.5-18.3	11.0 (7.9-14.1)	0.058
Loose stools	32.0 (28.5-35.5)	19.9 (16.1-23.7)	**<0.001**	30.8 (27.1-34.5)	20.2 (16.2-24.1)	**<0.001**
Use of antidiarrheal medication	14.7 (12.2-17.4)	10.9 (7.9-13.9)	0.084	15.2 (12.3-18.1)	10.4 (7.4-13.4)	**0.037**
Use of laxatives	11.6 (9.2-14.0)	21.4 (17.5-25.3)	**<0.001**	14.3 (11.5-17.1)	20.8 (16.8-24.8)	**0.001**

Prevalence of symptoms of bowel dysfunction grouped by sex

	1 year follow-up						*3 years follow-up*					
	Right-sided resection (%)			Left-sided resection (%)			Right-sided resection (%)			Left-sided resection (%)		
	Men	Women	p	Men	Women	p	Men	Women	p	Men	Women	p
Incontinence for flatus	19.5	28.9	**0.006**	27.1	35.7	0.075	15.7	23.5	**0.023**	17.8	31.2	**0.003**
Incontinence for stools	7.5	7.4	1.000	4.6	6.6	0.494	5.0	6.0	0.730	2.8	8.5	**0.023**
High frequency (>4/day)	7.6	7.2	0.944	10.9	8.8	0.589	7.9	6.2	0.543	7.5	5.3	0.504
Low frequency (<1/day)	25.6	24.4	0.790	21.8	22.7	0.938	27.9	24.6	0.421	30.8	30.4	1.000
Clustering	14.2	22.1	**0.011**	15.8	23.8	0.055	12.0	20.8	**0.006**	14.0	22.6	**0.037**
Urgency	11.4	21.4	**<0.001**	6.7	16.7	**0.002**	9.6	20.8	**<0.001**	7.9	14.8	**0.044**
Loose stools	28.4	35.4	0.060	17.1	23.6	0.123	28.2	33.2	0.215	16.9	24.2	0.095
Use of antidiarrheal medication	13.6	15.8	0.482	9.2	13.2	0.248	14.8	15.5	0.918	10.6	10.2	1.00
Use of laxatives	11.7	11.5	1.000	16.7	27.5	**0.011**	17.7	11.2	**0.033**	16.9	25.6	**0.047**
