# Supplementary material for: Bowel Dysfunction After Colon Cancer Surgery: A Prospective, Longitudinal, Multicenter Study
Source: Dis Colon Rectum. 2024 Jun 20;67(10):1322–31. doi: 10.1097/DCR.0000000000003358 (PMC11373893; doi:10.1097/DCR.0000000000003358)
Supplement: Supplementary file 4 [file dcr-67-1322-s004.pdf]

Supplement Table 3

To model the prevalence of LARS and distress, a generalized linear repeated measurements model
with a logit link was employed to account for the repeated measurements over time.

Major LARS

Fixed effects:

| | Estimate | Std. Error | z value | Pr(>\|z\|) | |
|---|---|---|---|---|---|
| (Intercept) | -0.6953 | 2.7481 | -0.253 | 0.8002 | |
| time | -1.9973 | 1.1463 | -1.742 | 0.0815 | . |
| Optyp_ny | -3.8432 | 2.0044 | -1.917 | 0.0552 | . |
| sex | -1.1373 | 1.6934 | -0.672 | 0.5018 | |
| age_groupyounger | -7.6763 | 4.7430 | -1.618 | 0.1056 | |
| Optyp_ny:sex | 2.0474 | 1.2262 | 1.670 | 0.0950 | . |
| Optyp_ny:age_groupyounger | 5.0428 | 3.0991 | 1.627 | 0.1037 | |
| sex:age_groupyounger | 4.0803 | 2.7953 | 1.460 | 0.1444 | |
| time:Optyp_ny | 1.5771 | 0.8178 | 1.929 | 0.0538 | . |
| time:sex | 0.9580 | 0.7054 | 1.358 | 0.1744 | |
| time:age_groupyounger | 2.8266 | 1.9407 | 1.456 | 0.1453 | |
| Optyp_ny:sex:age_groupyounger | -2.4232 | 1.8557 | -1.306 | 0.1916 | |
| time:Optyp_ny:sex | -0.7368 | 0.5002 | -1.473 | 0.1407 | |
| time:Optyp_ny:age_groupyounger | -1.8335 | 1.2614 | -1.453 | 0.1461 | |
| time:sex:age_groupyounger | -1.3494 | 1.1487 | -1.175 | 0.2401 | |
| time:Optyp_ny:sex:age_groupyounger | 0.7093 | 0.7606 | 0.933 | 0.3511 | |

Distress related to the bowel function, regardless of LARS score

Fixed effects:

| | Estimate | Std. Error | z value | Pr(>\|z\|) | |
|---|---|---|---|---|---|
| (Intercept) | -3.88844 | 2.32960 | -1.669 | 0.0951 | . |
| time | -0.51904 | 1.02791 | -0.505 | 0.6136 | |
| Optyp_ny | -0.01505 | 1.62356 | -0.009 | 0.9926 | |
| sex | 0.30359 | 1.44048 | 0.211 | 0.8331 | |
| age_groupyounger | 0.36199 | 3.90630 | 0.093 | 0.9262 | |
| Optyp_ny:sex | 0.37523 | 1.01710 | 0.369 | 0.7122 | |
| Optyp_ny:age_groupyounger | -0.33760 | 2.53912 | -0.133 | 0.8942 | |
| sex:age_groupyounger | 0.16343 | 2.35010 | 0.070 | 0.9446 | |
| time:Optyp_ny | 0.08297 | 0.72173 | 0.115 | 0.9085 | |
| time:sex | 0.51119 | 0.62814 | 0.814 | 0.4157 | |
| time:age_groupyounger | -0.11133 | 1.67947 | -0.066 | 0.9471 | |
| Optyp_ny:sex:age_groupyounger | -0.06192 | 1.55926 | -0.040 | 0.9683 | |
| time:Optyp_ny:sex | -0.21964 | 0.44380 | -0.495 | 0.6207 | |
| time:Optyp_ny:age_groupyounger | 0.39298 | 1.09267 | 0.360 | 0.7191 | |
| time:sex:age_groupyounger | -0.03135 | 1.00096 | -0.031 | 0.9750 | |
| time:Optyp_ny:sex:age_groupyounger | -0.15601 | 0.66523 | -0.235 | 0.8146 | |

---
