# Supplementary material for: Bowel Dysfunction After Colon Cancer Surgery: A Prospective, Longitudinal, Multicenter Study
Source: Dis Colon Rectum. 2024 Jun 20;67(10):1322–31. doi: 10.1097/DCR.0000000000003358 (PMC11373893; doi:10.1097/DCR.0000000000003358)
Supplement: Supplementary file 5 [file dcr-67-1322-s005.pdf]

Supplemental Table 4. Association between specific symptoms and distress

	Association with distress			Change of association over time		
Predictor	OR	95% CI	*p* value	OR	95% CI	*p* value
Incontinence for flatus	4.75	(2.04, 11.03)	**<0.001**	0.84	(0.57, 1.24)	0.38
Incontinence for stools	5.38	(1.28, 22.64)	**0.022**	1.11	0.58, 2.14)	0.75
High frequency (>4/day)	1.94	(0.52, 7.22)	0.32	1.27	(0.69, 2.34)	0.44
Low frequency (<1/day)	1.19	(0.43, 3.25)	0.74	1.16	(0.75, 1.79)	0.50
Clustering of stools	4.78	(1.61, 14.25)	**0.004**	0.54	(0.33, 0.91)	**0.02**
Urgency	2.23	(0.72, 6.87)	0.12	1.47	(0.88, 2.46)	0.14
Loose stools	7.00	(2.72, 18.04)	**<0.001**	0.67	(0.44, 1.02)	0.06
